# Comparative effects of minimally invasive approaches vs. conventional for obese patients undergoing aortic valve replacement: a systematic review and network meta-analysis

**DOI:** 10.1186/s12872-023-03410-9

**Published:** 2023-08-09

**Authors:** Shadi Alaa Abdelaal, Nadin Amr Abdelrahim, Mohamed Mamdouh, Nour Ahmed, Toka Reda Ahmed, Mahmoud Tarek Hefnawy, Latifa Kassem Alaqori, Mohamed Abozaid

**Affiliations:** 1 Kharkiv National Medical University, Kharkiv, Ukraine; 2 Faculty of Medicine, Tanta University, Tanta, Egypt; 3 Faculty of Medicine, Beni-Suef University, Beni-Suef, Egypt; 4 Faculty of Medicine, Zagazig University, Zagazig, Egypt; 5 Faculty of Medicine, University of Aden, Aden, Yemen; 6 Mayo Clinic, Rochester, MN USA

**Keywords:** Aortic valve replacement, Obesity, Minimally invasive approaches, Mini-sternotomy, Mini-thoracotomy

## Abstract

**Background:**

Minimally invasive approaches like mini-thoracotomy and mini-sternotomy for Aortic Valve Replacement (AVR) showed impressive outcomes. However, their advantages for obese patients are questionable. We aimed in this network meta-analysis to compare three surgical approaches: Full sternotomy (FS), Mini-sternotomy (MS), and Mini-thoracotomy (MT) for obese patients undergoing AVR.

**Methods:**

We followed the PRISMA extension for this network meta-analysis. PubMed/Medline, Scopus, Web of Science, and Cochrane searched through March 2023 for relevant articles. The analysis was performed using R version 4.2.3.

**Results:**

Out of 344, 8 articles met the criteria with 1392 patients. The main outcomes assessed were perioperative mortality, re-exploration, atrial fibrillation, renal failure, ICU stay, hospital stay, cross-clamp time, and bypass time. In favor of MS, the length of ICU stay and hospital stay was significantly lower than for FS [MD -0.84, 95%CI (-1.26; -0.43)], and [MD -2.56, 95%CI (-3.90; -1.22)], respectively. Regarding peri-operative mortality, FS showed a significantly higher risk compared to MS [RR 2.28, 95%CI (1.01;5.16)]. Also, patients who underwent minimally invasive approaches; MT and MS, required less need of re-exploration compared to FS [RR 0.10, 95%CI (0.02;0.45)], and [RR 0.33, 95%CI (0.14;0.79)], respectively. However, Intraoperative timings; including aortic cross-clamp, and cardiopulmonary bypass time, were significantly lower with FS than for MS [MD -9.16, 95%CI (-1.88; -16.45)], [MD -9.61, 95%CI (-18.64; -0.59)], respectively.

**Conclusion:**

Our network meta-analysis shows that minimally invasive approaches offer some advantages for obese patients undergoing AVR over full sternotomy. Suggesting that these approaches might be considered more beneficial alternatives for obese patients undergoing AVR.

**Supplementary Information:**

The online version contains supplementary material available at 10.1186/s12872-023-03410-9.

## Introduction

Obesity, according to the World Health Organization (WHO), is defined as an abnormal or excessive fat build-up that is linked to an increased risk of health issues, with a body mass index (BMI) of > 30 kg/m^2^ being considered obese. Moreover, obesity is a major public health issue worldwide and its prevalence has been increasing rapidly in recent decades [1, 2].

BMI elevation increases the risk of developing a large number of chronic illnesses including cardiovascular diseases, as well is considered one of the major risk factors for increased mortality. Obesity-related cardiovascular diseases are the major cause of mortality among obese accounting for 41%, as shown by epidemiologic studies [3, 4].

Studies have shown that cardiac surgeries for obese patients can result in significant reductions in mortality rates, despite the higher risks of complications associated with it such as acute renal failure and wound infections [5].

Minimally invasive cardiac surgeries have gained immense popularity over the past few decades owing to their ability to reduce complications, shorten hospital stays and provide faster recovery times [6, 7].

Following traditional cardiac surgeries, obesity has been associated with a greater risk of complications. Some studies have indicated that obesity is linked to an increased incidence of acute kidney injury and poor wound healing, whereas others have shown that obese patients are more likely to have longer hospitalizations and an increased risk of re-exploration [8–10]. Despite these risks and complications, minimally invasive approaches have emerged as an alternative option for obese patients for the purpose of minimizing invasiveness without compromising effectiveness and safety. Yet, it is debatable if these approaches are beneficial for obese patients in particular.

Therefore, we aimed in this systematic review and network meta-analysis to compare two minimally invasive approaches with the conventional one for obese aortic patients undergoing aortic valve surgeries.

## Methods

In accordance with the appropriate PRISMA extension [11], this systematic review and network meta-analysis was conducted.

**Search strategy.** A thorough and systematic search was conducted for literature in the PubMed, Scopus, Web of Science, and Cochrane databases. We applied the searches for studies published up to March 2023 using the following terms: (Sternotomy OR Partial Upper Sternotomy OR Mini-sternotomy OR Minimal Sternotomy OR J-shaped Sternotomy OR Full Sternotomy OR Conventional Sternotomy OR Median Sternotomy OR Mini-thoracotomy OR Standard Sternotomy) AND (Valve Replacement OR Valve Surgery OR Valve Implantation OR Surgical Valve Replacement OR Minimally Invasive Valve Surgery) AND (Obesity OR Central Obesity OR Abdominal Obesity OR Visceral Obesity OR Severe Obesity OR Morbid Obesity), with no applied language limitations. To ensure that no potentially relevant studies had been overlooked, we conducted a manual search into the references of systematic reviews related to this topic.

**Eligibility criteria and study selection.** Two reviewers screened titles, abstracts, and full texts of the databases’ retrieved articles in accordance with the eligibility criteria set out in the PICO framework; For obese patients undergoing aortic valve surgery (P), whether the use of minimally invasive approaches including; mini-sternotomy or mini-thoracotomy (I), compared to full sternotomy (C), favorably impact efficacy and prognostic outcomes including; perioperative mortality and re-exploration as primary outcomes, in addition, atrial fibrillation, renal failure, ICU stay, hospital stay, cross-clamp time, and bypass time as secondary outcomes (O). Studies to be included were either randomized controlled trials (RCTs) or observational cohort studies. If we didn’t have access to the full text or the study was done in vitro or on animals, it was excluded, Moreover, commentaries, conference abstracts, letters, and trial protocols were all excluded.

**Data extraction.** Two reviewers extracted data from the included studies using a standardized collection form that adhered to the guidelines set by the Cochrane Collaboration for Systematic Reviews [12]. The following data were extracted: first author, year of publication, sample size, number of males, mean BMI, mean age, perioperative mortality, re-exploration, hospital stay, ICU stay, cross-clamp time, bypass time, Renal failure, and atrial fibrillation. To resolve any differences in the extracted data, the two reviewers engaged in discussions and consulted with a senior investigator whenever required. This was done in a professional manner to ensure that all information was accurately recorded.

**Quality assessment.** Included studies’ risk of bias (ROB) was assessed using the Newcastle-Ottawa Scale (NOS) for cohort study quality evaluation [13].

**Statistical analyses.** A standard paired-wise meta-analysis was performed and the findings were presented as RR and MD, along with the corresponding 95% confidence intervals. The Cochrane’s Q test and I² statistic were used to evaluate the heterogeneity of the pooled data. The substantial heterogeneity was concluded when the P of the Q test was lower than 0.1, using DerSimonian-Laird random effects model.

The NMA was implemented using a random-effect model. Network plots were created showing; nodes that represent each intervention, and edges that reflect the number of studies in each comparison. A league table was used to display NMA results. The P-score ranking method was used to rank the interventions, indicating higher ranking suggested that the specified approach was more likely to be beneficial [14].

To check for inconsistencies between direct and indirect evidence, a node-splitting analysis was conducted and any value below 0.05 for P was deemed as an indication of such inconsistencies [15].

R software version 4.2.3 was used for generating all analyses and plots.

## Results

### Characteristics of the included studies and patients, and risk of bias

**Fig. 1:**
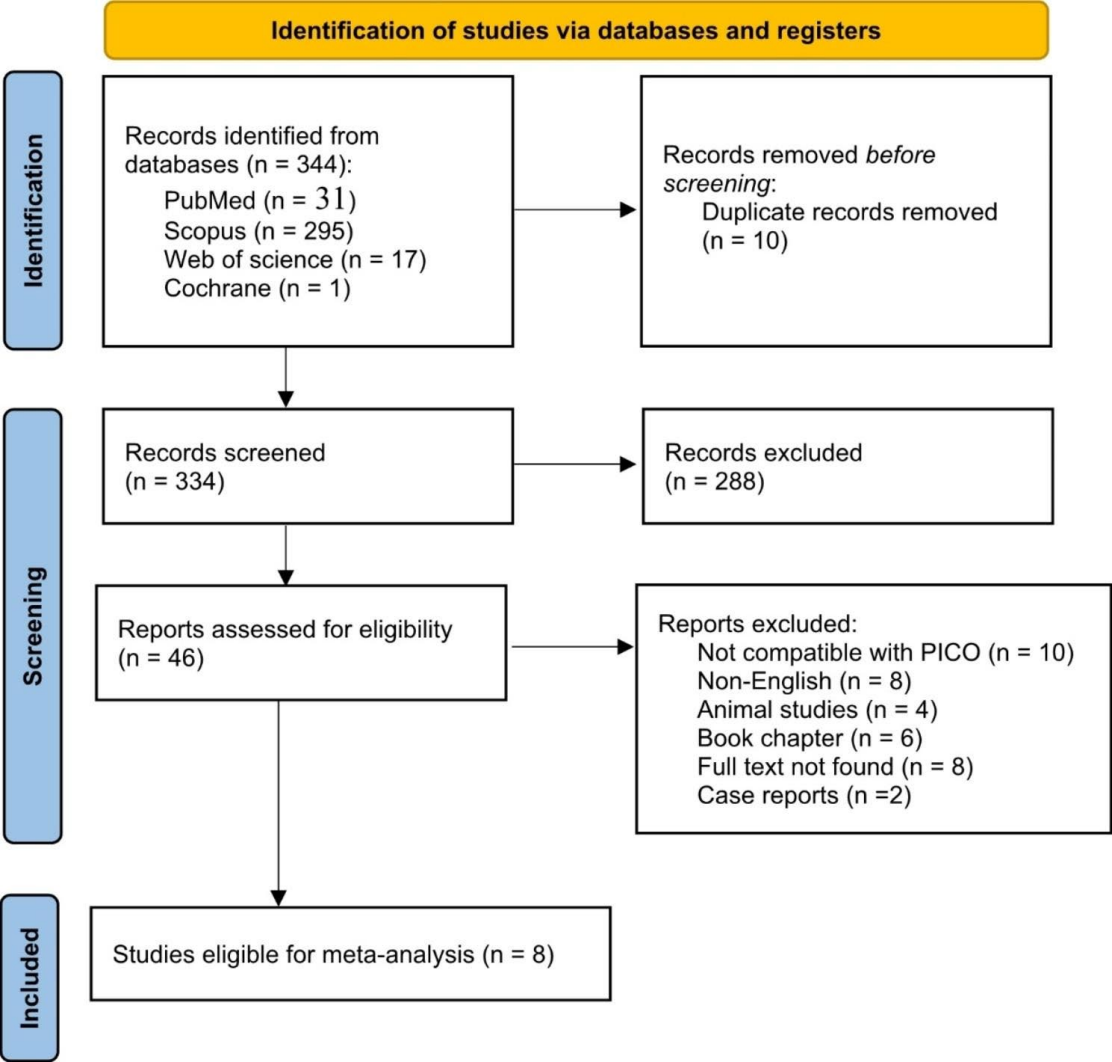
PRISMA flow diagram of the search for published trials showing search strategy with excluded studies and reason for exclusion

After screening and excluding ineligible articles (Fig. 1), 8 articles [16–23] aligned the inclusion criteria and provided relevant data for the statistical analysis. The baseline characteristics of patients are summarized in (Additional File 1: Table 1). A summary of the included studies’ properties is shown in (Additional File 1: Table 4).

The Newcastle-Ottawa scale was used to measure bias in the included studies, and every study received a 6 or higher (Additional File 1: Table 2).

### Pairwise meta-analysis

To obtain direct evidence, we conducted a pairwise meta-analyses of the available direct comparisons using a DerSimonian-Laird random effects model, the results are presented in Table 1. The pair-wise comparisons; mini-sternotomy vs. full sternotomy (MS vs. FS), and mini-thoracotomy vs. full sternotomy (MT vs. FS) had one or more studies, while the comparison; mini-sternotomy vs. mini-thoracotomy (MS vs. MT) had only one study.

**Table 1:** Pair-wise meta-analyses of articles directly comparing two types of approaches

Outcomes	Comparison	Studies	No. of Patients	Event 1	Event 2	RR/MD (95% CI)	P-value	Heterogeneity	
								I²	P
Perioperative mortality	MS vs. FS	5	974	8/543	17/431	**0.44 (0.19, 0.99)**	**< 0.05**	0.00	0.82
	MS vs. MT	1	437	3/271	1/166	1.84 (0.19, 17.52)	0.60	--	--
	MT vs. FS	1	342	1/166	8/176	0.13 (0.02, 1.05)	0.06	--	--
Renal failure	MS vs. FS	6	1138	54/625	68/513	0.75 (0.49, 1.16)	0.20	0.23	0.26
	MS vs. MT	1	437	23/271	12/166	1.17 (0.60, 2.3)	0.64	--	--
	MT vs. FS	2	390	14/187	25/203	0.61 (0.33, 1.14)	0.12	0.00	0.67
Atrial Fibrillation	MS vs. FS	3	748	127/439	81/309	1.07 (0.86, 1.33)	0.56	0.00	0.43
	MS vs. MT	1	437	117/271	62/166	1.16 (0.91, 1.47)	0.24	--	--
	MT vs. FS	2	390	65/187	72/203	0.96 (0.74, 1.25)	0.77	0.00	0.70
Re-exploration	MS vs. FS	4	824	16/477	44/347	**0.32 (0.14, 0.74)**	**< 0.05**	0.27	0.25
	MS vs. MT	1	437	11/271	2/166	3.37 (0.76, 15.01)	0.11	--	--
	MT vs. FS	2	390	2/187	38/203	**0.09 (0.02, 0.5)**	**< 0.05**	0.23	0.25
ICU stay	MS vs. FS	7	1178	645	533	**-0.79 (-1.19, -0.38)**	**< 0.05**	0.89	< 0.01
	MS vs. MT	1	437	271	166	**-0.33 (-0.48, -0.18)**	**< 0.05**	--	--
	MT vs. FS	2	390	187	203	-0.54 (-1.75, 0.68)	0.39	0.84	0.01
Hospital stay	MS vs. FS	7	1178	645	533	**-2.53 (-3.92, -1.13)**	**< 0.05**	0.92	< 0.01
	MS vs. MT	1	437	271	166	0.34 (-0.05, 0.73)	0.08	--	--
	MT vs. FS	2	390	187	203	-1.26 (-3.35, 0.84)	0.24	0.81	0.02
Cross-clamp time	MS vs. FS	7	1178	645	533	**8.79 (1.04, 16.55)**	**< 0.05**	0.93	< 0.01
	MS vs. MT	1	437	271	166	**11.17 (8.16, 14.18)**	**< 0.05**	--	--
	MT vs. FS	2	390	187	203	-5.64 (-19.02, 7.74)	0.39	0.92	< 0.01
Bypass time	MS vs. FS	7	1178	645	533	9.38 (-0.28, 19.04)	0.06	0.97	< 0.01
	MS vs. MT	1	437	271	166	**6.50 (2.89, 10.11)**	**< 0.05**	--	--
	MT vs. FS	2	390	187	203	-5.73 (-17.63, 6.17)	0.35	0.88	< 0.01

Statistically significant results are shown in bold; FS, full sternotomy; MT, mini-thoracotomy; MS, mini-sternotomy; CI, confidence interval; ICU, intensive care unit; MD, mean difference; RR, risk ratio

**Perioperative mortality.** Pairwise metanalysis between mini-sternotomy vs. mini-thoracotomy, and full sternotomy vs. mini-thoracotomy indicated no significant difference regarding the risk of perioperative mortality [RR 1.84, 95% CI (0.19, 17.52); 437 patients], [RR 0.13, 95% CI (0.02, 1.05); 342 patients], respectively. Conversely, there was metanalytical evidence of a significantly lower risk linked to full sternotomy shown by the comparison mini-sternotomy vs. full sternotomy [RR 0.44, 95% CI (0.19, 0.99); 5 studies, 974 patients] (Table 1.).

**Re-exploration.** In standard pairwise meta-analysis, compared with full sternotomy, both mini-sternotomy [RR 0.32, 95% CI (0.14, 0.74); 4 studies, 824 patients] and mini-thoracotomy [RR 0.09, 95% CI (0.02, 0.5); 2 studies, 390 patients] have been related to a reduced risk of re-exploration. However, a pairwise comparison of mini-sternotomy with mini-thoracotomy showed no statistically significant differences (Table 1.).

**Hospital stay.** Pairwise meta-analysis showed that mini-sternotomy was associated with shorter length of hospital stay than full sternotomy [MD -2.53, 95% CI (-3.92, -1.13); 7 studies, 1178 patients] with observed significant heterogeneity among these studies (I² = 92%, p < 0.001). Given the limited number of studies, meta-regression analysis couldn’t be conducted for identifying the potential sources of heterogeneity. Leave-one-out sensitivity analyses were applied and showed that two studies [23, 22] were the most contributors to the heterogeneity, however, their omission didn’t distort the effect estimate significance [MD -3.65, 95% CI (-4.46, -2.83)]. The other two pair-wise comparisons; mini-sternotomy vs. mini-thoracotomy, and mini-thoracotomy vs. full sternotomy, indicated no significant differences regarding hospital stay outcome (Table 1.).

**ICU stay.** Pairwise comparisons between mini-sternotomy vs. full sternotomy, and MS vs. mini-thoracotomy, revealed that mini-sternotomy was linked to a shorter duration of ICU stay compared to full sternotomy and mini-thoracotomy [MD -0.79, 95% CI (-1.19, -0.38); 7 studies, 1178 patients], [MD -0.33 95% CI (-0.48, -0.18); 1 study, 437 patients], respectively. There was a significant heterogeneity observed among studies comparing mini-sternotomy with full sternotomy (I² = 89%, p < 0.001). Two studies [23, 22] were shown to have contributed the most to the observed heterogeneity through the use of Leave-one-out sensitivity analyses, without a distortion of the effect estimate significance by their exclusion [MD -1.28, 95% CI (-1.60, -0.96)]. Comparison of mini-thoracotomy with full sternotomy showed no significant difference regarding the duration of ICU stay (Table 1.).

**Cross-clamp time.** As shown by pairwise meta-analyses of mini-sternotomy vs. full sternotomy and mini-sternotomy vs. mini-thoracotomy, mini-sternotomy was associated with a longer cross-clamp time compared to full sternotomy and mini-thoracotomy [MD 8.79, 95% CI (1.04, 16.55); 7 studies, 1178 patients], [MD 11.17 95% CI (8.16, 14.18); 1 study, 437 patients], respectively. Heterogeneity among studies comparing mini-sternotomy with full sternotomy was significant (I² = 93%, p < 0.001). leave-one-out sensitivity analyses indicated that these two studies [22, 21] were the major causes of heterogeneity, nevertheless, their removal did not affect the effect estimate significance [MD 7.26, 95% CI (4.33, 10.18)] (Table 1.).

**Bypass time.** According to pairwise meta-analyses shown significance, mini-sternotomy was associated with a longer bypass time compared to mini-thoracotomy [MD 6.50, 95% CI (2.89, 10.11); 1 study, 437 patients]. Pairwise meta-analysis of studies comparing mini-sternotomy with full sternotomy revealed substantial heterogeneity (I² = 97%, p < 0.001). Three studies [23, 22, 21] were found to be the key sources of heterogeneity as indicated by leave-one-out sensitivity analysis (I²= 2%, P = 0.38). Remarkably, their omission had an impact on the pooled effect estimate significance, revealing that a longer bypass time was linked to mini-sternotomy comparing to full sternotomy [MD 4.44, 95% CI (0.47, 8.41)] (Table 1.).

**Other outcomes.** Pairwise meta-analyses of the outcomes including atrial fibrillation and renal failure revealed no significant differences. The pooled results in these pairwise comparisons showed low heterogeneity (Table 1.)

### Network meta-analysis

Network meta-analysis findings are shown in Table 2. and P-score ranking values are presented in Table 3. All estimates for each outcome were derived from both direct and indirect evidence. The major part of the direct comparisons was between mini-sternotomy and full sternotomy for all of the measured outcomes.

**Table 2:** League table with network meta-analytic estimates

Outcome	Full Sternotomy	0.48 (0.22; 1.04)	0.25 (0.04; 1.52)	Mortality; [RR]
		0.78 (0.54; 1.13)	0.65 (0.34; 1.22)	**Renal Failure; [RR]**
		1.07 (0.86; 1.34)	0.94 (0.73; 1.23)	**Atrial Fibrillation; [RR]**
		0.33 (0.14; 0.79) **	0.10 (0.02; 0.45) **	**Re-exploration; [RR]**
		-0.84 (-1.26; -0.43) **	-0.51 (-1.17; 0.14)	**ICU stay; [MD]**
		-2.56 (-3.90; -1.22) **	-1.95 (-4.05; 0.14)	**Hospital stay; [MD]**
		**9.16 (1.88; 16.45)**	-3.57 (-16.28; 9.14)	**Cross-clamp time; [MD]**
		**9.61 (0.59; 18.64)**	-2.13 (-17.79; 13.52)	**Bypass time; [MD]**
**Mortality; [RR]**	**2.28 (1.01; 5.16)**	**Mini-sternotomy**	0.35 (0.04; 2.96)	**Mortality; [RR]**
**Renal Failure; [RR]**	1.28 (0.89; 1.86)		0.83 (0.43; 1.59)	**Renal Failure; [RR]**
**Atrial Fibrillation; [RR]**	0.93 (0.75; 1.16)		0.88 (0.69; 1.11)	**Atrial Fibrillation; [RR]**
**Re-exploration; [RR]**	**3.00 (1.27; 7.08)**		0.31 (0.06; 1.48)	**Re-exploration; [RR]**
**ICU stay; [MD]**	**0.84 (0.43; 1.26)**		0.33 (-0.35; 1.01)	**ICU stay; [MD]**
**Hospital stay; [MD]**	**2.56 (1.22; 3.90)**		0.61 (-1.63; 2.84)	**Hospital stay; [MD]**
**Cross-clamp time; [MD]**	-9.16 (-16.45; -1.88) **		-12.73 (-26.24; 0.78)	**Cross-clamp time; [MD]**
**Bypass time; [MD]**	-9.61 (-18.64; -0.59) **		-11.75 (-28.43; 4.94)	**Bypass time; [MD]**
**Mortality; [RR]**	6.46 (0.83; 50.14)	2.83 (0.34; 23.66)	**Mini-thoracotomy**	**Outcome**
**Renal Failure; [RR]**	1.55 (0.82; 2.93)	1.21 (0.63; 2.32)		
**Atrial Fibrillation; [RR]**	1.06 (0.82; 1.38)	1.14 (0.90; 1.44)		
**Re-exploration; [RR]**	**9.82 (2.21; 43.64)**	3.27 (0.68; 15.83)		
**ICU stay; [MD]**	0.51 (-0.14; 1.17)	-0.33 (-1.01; 0.35)		
**Hospital stay; [MD]**	1.95 (-0.14; 4.05)	-0.61 (-2.84; 1.63)		
**Cross-clamp time; [MD]**	3.57 ( -9.14; 16.28)	12.73 ( -0.78; 26.24)		
**Bypass time; [MD]**	2.13 (-13.52; 17.79)	11.75 ( -4.94; 28.43)		

The number in each cell refers to the comparison between the given column and row, The order of treatments in the diagonal is arbitrary and does not reflect ranking, Data are shown as RR/MD and 95% confidence intervals, **Marks significant effect estimates in a given cell in which favors the column-defining treatment, Bold font denotes significant effect estimates in a given cell in which favors the row-defining treatment, ICU; Intensive care unit, RR; Risk ratio, MD; Mean difference

**Table 3:** P-score ranking values of different interventions and outcomes were analyzed

	Perioperative mortality	Renal failure	Atrial Fibrillation	Re-exploration	ICU stay	Hospital stay	Cross-clamp time	Bypass time
FS	0.030	0.092	0.204	0.004	0.031	0.017	0.642	0.688
MS	0.573	0.596	0.534	0.532	0.915	0.851	0.020	0.051
MT	0.897	0.812	0.762	0.964	0.555	0.632	0.838	0.761

FS, Full Sternotomy; MT, Mini-thoracotomy; MS, Mini-sternotomy; ICU, Intensive care unit

**Network consistency.** The direct and indirect evidence were consistent with each other; as node-splitting analysis did not reveal significant inconsistency between direct and indirect evidence for any outcomes; P > 0.05 (Additional File 1: Table 3).

**Re-exploration.** NMA showed that the risk of re-exploration was significantly higher for full sternotomy than for mini-sternotomy [RR 3.00, 95% CI 1.27, 7.08] and mini-thoracotomy [RR 9.82, 95% CI 2.21, 43.64]. However, there was a similar risk for mini-thoracotomy as for mini-sternotomy [RR 0.31, 95% CI 0.06, 1.48]. The p-score ranking method ranked mini-thoracotomy higher and full sternotomy lower, which encourages the NMA findings; mini-thoracotomy vs. full sternotomy [RR 0.10, 95% CI 0.02, 0.45], mini-sternotomy vs. full sternotomy [RR 0.33 95% CI 0.14, 0.79] (Table 2.) (Table 3.).

**ICU stay.** As shown by NMA, full sternotomy required more time in ICU compared to mini-sternotomy [MD 0.84, 95% CI (0.43; 1.26)]. In parallel to that, the P-score ranking method assigned a higher ranking to mini-sternotomy and a lower to full sternotomy. NMA showed a non-significant difference in ICU duration for mini-thoracotomy when compared to mini-sternotomy and full sternotomy [MD 0.33, 95% CI (-0.35; 1.01)], [MD -0.51, 95% CI (-1.17; 0.14)], respectively (Table 2.) (Table 3.).

**Hospital stay.** Based on NMA findings, full sternotomy had a significantly longer hospital stay than mini-sternotomy [MD 2.56, 95% CI (1.22; 3.90)]. Likewise, the P-score ranking method gave the highest ranking to mini-sternotomy whereas full sternotomy was the lowest. Comparing mini-thoracotomy to full sternotomy, and mini-sternotomy; the difference in hospital stay duration was not statistically significant [MD -1.95, 95% CI (-4.05; 0.14)] [MD 0.61, 95% CI (-1.63; 2.84)], respectively (Table 2.) (Table 3.).

**Perioperative mortality.** With full sternotomy as compared to mini-sternotomy, NMA revealed that the risk of perioperative mortality was significantly higher in full sternotomy [RR 2.28, 95%CI (1.01; 5.16)]. Even though the P-score ranking method placed mini-thoracotomy higher than other approaches, NMA results regarding the risk of perioperative mortality did not substantially favor mini-thoracotomy over the other techniques [RR 0.35, 95%CI (0.04; 2.96)] [RR 0.25, 95% CI (0.04; 1.52)], respectively (Table 2.) (Table 3.).

**Cross clamp time.** According to NMA results, cross-clamp time in full sternotomy was significantly shorter than compared to mini-sternotomy [MD -9.16, 95%CI (-16.45; -1.88)]. The P-score ranking method assigned the highest ranking to mini-thoracotomy and the lowest to mini-sternotomy, nonetheless, cross-clamp time in mini-thoracotomy was not statistically significant when compared to mini-sternotomy and full sternotomy [MD -12.73, 95% CI (-26.24; 0.78)], [ MD -3.57 95% CI (-16.28; 9.14)], respectively (Table 2.) (Table 3.).

**Bypass time.** Bypass time was significantly shorter in full sternotomy than in mini-sternotomy, as shown by NMA [MD -9.61, 95% CI (-18.64; -0.59)]. Despite the P-score ranking method ranked mini-thoracotomy over other approaches, NMA findings did not significantly favor mini-thoracotomy over mini-sternotomy or full sternotomy [MD -11.75, 95% CI (-28.43; 4.94)], [ MD -2.13 95% CI (-17.79; 13.52)], respectively (Table 2.) (Table 3.).

**Other outcomes.** As shown by NMA, the three types of approaches have no significant differences regarding the risk of atrial fibrillation neither nor renal failure. P-score method ranked mini-thoracotomy first and full sternotomy last for lower risk of renal failure. While also it raked mini-thoracotomy first, mini-sternotomy was ranked last for lower risk of atrial fibrillation. (Table 2.) (Table 3.).

## Discussion

Obese patients as defined by having a BMI of 30 kg/m^2^ or higher, are at risk of developing cardiovascular diseases and several other health problems that are associated with the condition, including dyslipidemia, hypertension, diabetes, and insulin resistance [24]. Obesity also was linked to an increased occurrence of deep sternal wound infection after cardiac surgery, as well as other complications, including wound reopening, the need for prolonged ventilation, and having an extended duration of hospital stay [9, 25]. Nonetheless, some studies have indicated that obese patients may have lower mortality rates and lower chances of bleeding after cardiac surgery [26, 27]. This could be attributed to their selection for surgeries with a lower risk of bias and confounding factors [28]. This adds more to the paradox of selecting the appropriate surgical approach for obese patients undergoing surgeries for AVR.

To our knowledge, this is the first network meta-analysis to investigate the clinical outcomes of obese patients who have undergone aortic valve replacement using three different surgical approaches: mini-sternotomy, mini-thoracotomy, and full sternotomy.

The clinical usage of minimally invasive methods for AVR such as; mini sternotomy and mini-thoracotomy, was first documented for clinical use in 1996 [29]. Since then, there have been conflicting perspectives on whether these techniques are more advantageous than conventional ones or not. In a study conducted by Furukawa et al. on 984 elderly patients who underwent either conventional sternotomy or mini-sternotomy, their findings suggested that using mini sternotomy for AVR surgery was safe and with lower risk of complications, but the majority of participants in this study weren’t obese, having a BMI of 27 kg m2 or lower [30].

Recent meta-analyses have indicated that minimally invasive approaches are a safe choice for patients undergoing AVR when compared to conventional ones. Even so, some even reported that minimal access AVR may take longer with cross-clamp and bypass duration. However, due to the limitations, these analyses did not identify significant and robust evidence for deciding upon the appropriate approach [31–33].

For obese patients undergoing AVR using minimally invasive approaches, our results which combine the clinical findings from the recently published literature showed that minimally invasive interventions; such as mini-sternotomy, were safe and generally favored over full sternotomy for having better significant outcomes as well as re-exploration, perioperative mortality, hospital, and ICU stays. However, our findings along with literature [34–36] showed that mini-sternotomy was associated with longer Cross-clamp time and bypass time compared with full sternotomy; this may limit the choice of mini-sternotomy intervention with certain patients but have no impact on the other clinical outcome or postoperative infection.

As regards perioperative mortality, our results showed that mini-sternotomy was favored over full-sternotomy and mini-thoracotomy, supporting that of Santana et al. [37] In which minimally invasive approaches have lower mortality and morbidity compared with full sternotomy. Still, several studies [38–40] reported no significant differences in mortality or morbidity between patients undergoing minimal access and conventional AVR. This may be explained by the fact that low-risk and young patients usually request Minimal invasive interventions for cosmetic reasons and a shorter recovery duration.

Mini-thoracotomy is a common alternative approach for mini sternotomy that has been reported in our study [22, 19] and literature [41, 42] to have advantages over full-sternotomy. It is also worth noting that there are limited publications directly comparing mini-thoracotomy and mini-sternotomy; our network findings generally favored mini-sternotomy over mini-thoracotomy supporting the conclusions of Balmforth et al. [43] which found no significant benefits of mini-thoracotomy over mini sternotomy; however, mini-thoracotomy was favorable over mini-sternotomy in terms of re-exploration making it more suitable intervention for patients having a higher risk of re-exploration.

Since the mini-thoracotomy approach has more exclusion criteria based on anatomy, it may not be routinely offered to obese patients. Besides that, the procedure is also more technically challenging and takes longer compared to the mini-sternotomy, so the choice between both interventions may be determined according to each patient’s characteristics.

## Limitations

This network meta-analysis; comparing three surgical approaches for obese patients undergoing AVR had some limitations. First, the studies included in the analysis were not randomized controlled trials, some had small sample sizes and had varying patients’ characteristics. Furthermore, the long-term outcomes of these approaches were not evaluated.

However, despite these limitations, the study still provides valuable information to guide clinical decision-making for obese patients undergoing AVR.

## Recommendations

Surgeons and healthcare providers should carefully consider the individual patient’s specific needs and risk factors when selecting an approach for obese patients undergoing AVR. Taking into consideration the patient’s aortic anatomical landmarks with the sternum, as well as the patient’s history of pleural conditions; as they are crucial elements influencing the accuracy of choosing the optimal approach.

Also, it is necessary to conduct additional clinical trials to assess the long-term outcomes and cost-effectiveness of the various surgical approaches for obese patients undergoing AVR.

## Conclusion

Our network meta-analysis findings showed that minimally invasive approaches for obese patients undergoing AVR have advantages over the conventional full sternotomy in terms of reduced risk of re-exploration, shorter hospital stay, and lower risk of perioperative mortality. However, full sternotomy had shorter cross-clamp and bypass times, necessitating patients’ selection for these minimally invasive approaches. The three approaches did not differ significantly regarding the risk of atrial fibrillation or renal failure.

Overall, our findings led us to the conclusion that minimally invasive approaches might be considered more beneficial alternatives for obese patients undergoing AVR.

### Electronic supplementary material

Below is the link to the electronic supplementary material.

Additional File 1: Supplemental Tables and Figures

## Data Availability

The datasets used and/or analysed are available from the corresponding author on reasonable request.

## Abbreviations

AVR: Aortic Valve Replacement
MS: Mini-sternotomy
MT: Mini-thoracotomy
FS: Full Sternotomy
WHO: World Health Organization
NMA: Network meta-analysis
NOS: Newcastle-Ottawa Scale
ROS: Risk of bias
BMI: Body mass index
CI: Confidence interval
MD: Mean difference
RR: Risk ratio
ICU: Intensive care unit
