# Supplementary material for: Comparative effects of minimally invasive approaches vs. conventional for obese patients undergoing aortic valve replacement: a systematic review and network meta-analysis
Source: BMC Cardiovasc Disord. 2023 Aug 9;23:392. doi: 10.1186/s12872-023-03410-9 (PMC10413702; doi:10.1186/s12872-023-03410-9)
Supplement: Supplementary file 1 — Additional File 1: Supplemental Tables and Figures [file 12872_2023_3410_MOESM1_ESM.docx]

**Table 2. Quality of the observational cohort studies, based on the Newcastle - Ottawa scale**..........

13

**Figure 8. NMA of renal failure**...................................................................................................

12

**Figure 7. NMA of re-exploration**...............................................................................................

11

**Figure 6. NMA of peri-operative mortality**...............................................................................

9

**Figure 4. NMA of hospital stay**..................................................................................................

8

**Figure 3. NMA of cross-clamp time**...........................................................................................

7

**Figure 2. NMA of bypass time**....................................................................................................

10

**Figure 5. NMA of ICU stay**.........................................................................................................

6

**Figure 1. NMA of atrial fibrillation**...........................................................................................

5

**Table 4. Summary of included studies properties and results**.................................................

4

**Table 3. Node-splitting analysis of direct and indirect evidence**.............................................

3

2

**Table 1. Characteristics of the studies in the network meta-analysis**.....................................

**CONTENT**

***Supplementary*** ***Table 1. Characteristics of the studies in the network meta-analysis.***

| Author | Year | Intervention | No. of  Patients | Mean BMI (kg/m2) | Male (n) | Mean age (year) | HTN (n) | DM (n) | Mean EuroSCORE II | Mean EF (%) | Study type |
| --- | --- | --- | --- | --- | --- | --- | --- | --- | --- | --- | --- |
| Abud et al. | 2022 | FS | 27 | 36.18 ± 4.65 | -- | 71.22 ± 6.14 | -- | 13 | 3.28 ± 2.17 | 45.3 ± 23.5 | Cohort study |
|  |  | MT | 21 | 36.39 ± 5.21 | -- | 69.43 ± 7.11 | -- | 12 | 3.76 ± 2.15 | 43.3 ± 19.9 |  |
| Aliahmed et al. | 2017 | FS | 56 | 30.34 ± 4.15 | 38 | 61.92 ± 14.16 | -- | -- | -- | -- | Cohort study |
|  |  | MS | 56 | 29.61 ± 3.84 | 36 | 62.08 ± 10.55 | -- | -- | -- | -- |  |
| Girgis et al. | 2022 | FS | 20 | 41.63 ± 1.25 | 10 | 37.65 ± 8.25 | 5 | 10 | -- | 58.1 ± 7.06 | Cohort study |
|  |  | MS | 20 | 41.78 ± 1.44 | 11 | 36.8 ± 9.3 | 8 | 10 | -- | 59.65 ± 6.66 |  |
| Luo et al. | 2022 | FS | 88 | 30.11 ± 3.04 | 70 | 55.9 ± 10.8 | 79 | 16 | -- | 61.7 ± 8.9 | Cohort study |
|  |  | MS | 70 | 30.56 ± 3.09 | 55 | 54.0 ± 11.5 | 63 | 12 | -- | 62.5 ± 10.2 |  |
| Mikus et al. | 2020 | FS | 176 | 32.2 ± 2.2 | 82 | 72.7 ± 8.2 | 134 | 55 | 7.01 ± 1.5 | 59.7 ± 6.7 | Cohort study |
|  |  | MS | 271 | 32.8 ± 2.5 | 145 | 74.3 ± 7.5 | 203 | 64 | 6.7 ± 2.2 | 60 ± 7.5 |  |
|  |  | MT | 166 | 32.6 ± 2.8 | 86 | 72.6 ± 8.8 | 129 | 50 | 6.0 ± 1.5 | 62 ± 8.23 |  |
| Pisano et al. | 2017 | FS | 42 | 29.5 ± 8.5 | 21 | 76.5 ± 61.1 | 37 | 3 | 6.4 ± 3.8 | -- | Cohort study |
|  |  | MS | 42 | 30.4 ± 6.1 | 15 | 76 ± 33.1 | 30 | 9 | 5.0 ± 4.6 | -- |  |
| Welp et al. | 2018 | FS | 91 | 33.11 ± 3.04 | 51 | 70.02 ± 10.51 | 87 | 35 | 7.96 ± 7.48 | 58.33 ± 10.82 | Cohort study |
|  |  | MS | 126 | 32.56 ± 3.09 | 69 | 69.79 ± 10.38 | 106 | 36 | 8.58 ± 8.78 | 55.16 ± 14.90 |  |
| Xie et al. | 2022 | FS | 60 | 35.9 ± 4.5 | 32 | 60.1 ± 10.9 | 46 | 6 | 7.0 ± 6.1 | 59 ± 9.9 | Cohort study |
|  |  | MS | 60 | 34.9 ± 5.1 | 28 | 60.6 ± 11.3 | 43 | 5 | 7.0 ± 6.1 | 60.3 ± 8.3 |  |

BMI, Body Mass Index; HTN, Hypertension; DM, Diabetes Mellitus; EuroSCORE, European System for Cardiac Operative Risk Evaluation; EF, Ejection Fraction; FS, Full Sternotomy; MT, Mini-thoracotomy; MS, Mini-sternotomy.

***Supplementary*** ***Table 2. Quality of the observational cohort studies, based on the Newcastle–Ottawa scale.***

| Study | Selection | | | | Comparability | Outcome | | | Score |
| --- | --- | --- | --- | --- | --- | --- | --- | --- | --- |
|  | **I** | **II** | **III** | **IV** | **V** | **VI** | **VII** | **VIII** |  |
| Abud et al. 2022 | * | * | * | -- | ** | -- | * | -- | 6 |
| Aliahmed et al. 2017 | * | * | * | -- | ** | * | -- | -- | 6 |
| Girgis et al. 2022 | * | * | -- | * | ** | * | -- | -- | 6 |
| Luo et al. 2022 | * | * | * | * | ** | * | -- | -- | 7 |
| Mikus et al. 2020 | * | * | * | * | ** | * | -- | -- | 7 |
| Pisano et al. 2017 | * | * | -- | * | ** | * | -- | -- | 6 |
| Welp et al. 2018 | * | * | * | * | ** | * | -- | -- | 7 |
| Xie et al. 2022 | * | * | * | * | ** | * | -- | -- | 7 |

I, Representativeness of the exposed cohort; II, Selection of the non-exposed cohort; III, Ascertainment of exposure; IV, Demonstration that the outcome of interest was not present at start of study; V, Comparability of cohorts on the basis of the design or analysis; VI, Assessment of outcome; VII, Was follow-up long enough for outcomes to occur?; VIII, Adequacy of cohort follow-up.

***Supplementary*** ***Table 3. Node-splitting analysis of direct and indirect evidence.***

| **Outcome** | **Comparison** | **K** | **Prop** | **NMA** | **Direct** | **Indirect** | **Difference** | **P-value** |
| --- | --- | --- | --- | --- | --- | --- | --- | --- |
| Perioperative mortality | MS vs. FS | 5 | 1 | 0.44 | 0.44 | -- | -- | -- |
|  | MS vs. MT | 1 | 0.89 | 2.83 | 1.84 | 85.21 | 0.02 | 0.26 |
|  | MT vs. FS | 1 | 0.98 | 0.15 | 0.13 | 986.67 | < 0.01 | 0.26 |
| Renal failure | MS vs. FS | 6 | 0.99 | 0.78 | 0.77 | 1.49 | 0.52 | 0.73 |
|  | MS vs. MT | 1 | 0.79 | 1.21 | 1.74 | 1.34 | 0.88 | 0.87 |
|  | MT vs. FS | 2 | 0.90 | 0.65 | 0.61 | 1.01 | 0.61 | 0.65 |
| Atrial Fibrillation | MS vs. FS | 3 | 0.99 | 1.07 | 1.07 | 1.75 | 0.61 | 0.68 |
|  | MS vs. MT | 1 | 0.98 | 1.14 | 1.16 | 0.52 | 2.21 | 0.35 |
|  | MT vs. FS | 2 | 0.98 | 0.94 | 0.96 | 0.4 | 2.41 | 0.34 |
| Re**-**exploration | MS vs. FS | 4 | 0.99 | 0.33 | 0.32 | 27.35 | 0.01 | 0.34 |
|  | MS vs. MT | 1 | 0.79 | 3.27 | 3.37 | 2.94 | 1.14 | 0.95 |
|  | MT vs. FS | 2 | 0.97 | 0.1 | 0.09 | 27.05 | < 0.01 | 0.23 |
| ICU stay | MS vs. FS | 7 | 0.98 | -0.84 | -0.80 | -2.76 | 1.96 | 0.18 |
|  | MS vs. MT | 1 | 0.66 | -0.33 | -0.33 | -0.33 | -0.01 | 0.99 |
|  | MT vs. FS | 2 | 0.86 | -0.51 | -0.39 | -1.25 | 0.86 | 0.37 |
| Hospital stay | MS vs. FS | 7 | 0.97 | -2.56 | -2.52 | -4.24 | 1.73 | 0.69 |
|  | MS vs. MT | 1 | 0.58 | -0.61 | 0.34 | -1.89 | 2.23 | 0.33 |
|  | MT vs. FS | 2 | 0.87 | -1.95 | -1.28 | -6.64 | 5.37 | 0.10 |
| Cross-clamp time | MS vs. FS | 7 | 0.98 | 9.16 | 8.80 | 25.96 | -17.16 | 0.51 |
|  | MS vs. MT | 1 | 0.52 | 12.73 | 11.17 | 14.43 | -3.27 | 0.81 |
|  | MT vs. FS | 2 | 0.87 | -3.75 | -5.63 | 10.53 | -16.16 | 0.41 |
| Bypass time | MS vs. FS | 7 | 0.98 | 9.61 | 9.41 | 19.04 | -9.63 | 0.76 |
|  | MS vs. MT | 1 | 0.52 | -2.13 | 6.50 | 17.34 | -10.84 | 0.52 |
|  | MT vs. FS | 2 | 0.88 | 11.75 | -5.58 | 22.16 | -27.74 | 0.25 |

K, Number of studies providing direct evidence; NMA, Estimated treatment effect (RR/MD) in network meta-analysis; Prop, Proportion of direct evidence; Direct, Estimated treatment effect (RR/MD) derived from direct evidence; Indirect, Estimated treatment effect (RR/MD) derived from indirect evidence; Difference, difference in the network (direct versus indirect); P-value, p-value of test for disagreement (direct versus indirect). P＜0.05 was considered to indicate inconsistency and was shown in bold. ICU, intensive care unit; FS, Full Sternotomy; MT, Mini-thoracotomy; MS, Mini-sternotomy.

***Supplementary*** ***Table 4. Summary of included studies properties and results.***

| **Study** | **Country** | **Intervention** | **Inclusion criteria** | **Exclusion criteria** | **Results** |
| --- | --- | --- | --- | --- | --- |
| Abud et al. 2022 | Turkey | FS | Obese patients  with isolated severe aortic valve stenosis or regurgitation. | History of right pleurisy or pleural effusion with adhesion formation, chest wall deformities, previous major cardiac surgery involving pericardium opening, right-sided thoracic surgery, porcelain aorta, aneurysmal  dilatation or ascending aortic dissection (≥4.5 cm) and concomitant significant coronary artery stenosis or other valve disease. | Mean cross-clamp and cardiopulmonary bypass times were  similar in both groups. Operating time was significantly longer in the minimally invasive group (P <0.05). In the minimally invasive group, acute renal failure occurred in 2 patients. |
|  |  | MT |  |  |  |
| Aliahmed et al. 2017 | Lithuania | FS | Obese patients underwent an isolated replacement of the  aortic valve by sternotomy or mini sternotomy at Vilnius University Hospital, Lithuania in the period from 2011 to 2016. | -- | Mini sternotomy had longer cross clamp 91 ± 20 min vs 76 ± 23 min (p < 0.001). and bypass times 148 ± 4 min vs 133 ± 2 min (р = 0,023). In the mini sternotomy group acute renal failure developed less frequently – 2 pts. (3.6%) vs 12 pts. (21.43%) (p = 0.004). No significant differences found in the hospital stay, intensive care unit stay. |
|  |  | MS |  |  |  |
| Girgis et al. 2022 | Egypt | FS | Morbid obese patients (body mass index ≥ 40) who had undergone isolated AVR surgery at the National Heart Institute from June 2019 to June 2022. | Patients with concomitant cardiac conditions, redo cases and pre-operative comorbidities (hepatic, renal, cerebrovascular …etc.). | Patients treated with mini sternotomy AVR had presented advantages in terms of shorter intensive care unit and hospital stay (p= 0.0001). However, the operative, cross-clamp, and bypass times were significantly longer (p= 0.0001) in the minimally invasive group. |
|  |  | MS |  |  |  |
| Luo et al. 2022 | China | FS | Obese and non-obese aortic patients that underwent treatment via a PUS to those of patients that were treated using a conventional FMS approach at Medical University Union Hospital, China from January 2015 to January 2021. | Patients were excluded if they: (1) had been diagnosed with aortitis, Marfan syndrome, metoxoarteritis, or systemic immune disorders, or (2) had previously undergone organ transplantation or experienced infective endocarditis, cardiogenic shock, malignancies, or chronic organ failure. | There were no differences between the full median sternotomy and partial upper sternotomy groups with respect to preoperative baseline indicators and postoperative complications. The partial upper sternotomy approach was associated with shorter intensive care unit stay (P= 0.001), shorter duration of hospitalization (P= 0.010). |
|  |  | MS |  |  |  |
| Mikus et al. 2020 | Italy | FS | Obese patients underwent isolated AVR at Maria Cecilia Hospital, Italy from January 2010 to November 2019. | The only exclusion criterion for right mini-thoracotomy was the history of previous left pneumectomy. There were no contraindications for partial or standard full sternotomy. | Patients treated with minimally invasive approaches had shorter cardiopulmonary bypass time (p = .012) and aortic cross‐clamp time (p = .022). |
|  |  | MS |  |  |  |
|  |  | MT |  |  |  |
| Pisano et al. 2017 | Italy | FS | Obese patients undergoing isolated primary AVR with  standard prosthesis for a 2-year period. | -- | Cardiopulmonary bypass time was significantly longer in MS group compared with the FS group [median (95% confidence level or CL), 103 (98.7–106.4) vs 94 (83.6–99) minutes, respectively; P = 0.0019]. No significant difference  was observed in aortic cross-clamp time [median (95% CL), 73 (71.1–78.2) vs 69.5 (62.7–83) minutes; P = 0.4]. Significantly shorter intensive care unit stay [median (95% CL), 1 vs 2 days; P = 0.00017], and hospital stay [median (95% CL), 8.5 (8–10.8) vs 13.5 (11.1–14) days; P = 0.00030] were shown in the MS group. |
|  |  | MS |  |  |  |
| Welp et al. 2018 | Germany | FS | Obese patients who underwent isolated AVR for the first time between January 2000 and January 2015 at University Hospital Mu¨nster. | -- | Mortality and renal failure were equivalent. Mini-AVR was associated with shorter intensive care unit (ICU) stay  [2 days (min 1 day; max 25 days) vs 4 days (min 1 day; max 35 days); P = 0.031]. Total duration of hospital stay was comparable. |
|  |  | MS |  |  |  |
| Xie et al. 2022 | China | FS | - Preoperative BMI≥30 kg m2.  - Age≥18 years  - No previous cardiac surgery  - Non-emergency operation  - Left ventricular ejection fraction > 30%. | -- | No in‑hospital death occurred in the two groups. In addition, cardiopulmonary bypass time was similar across the  2 groups, but the aortic cross‑clamp time was significantly shorter in the con‑AVR group (P=.0.022). There was a shorter intensive care unit stay time (P= 0.021) in the mini‑AVR group. |
|  |  | MS |  |  |  |

FS, Full Sternotomy; MT, Mini-thoracotomy; MS, Mini-sternotomy.


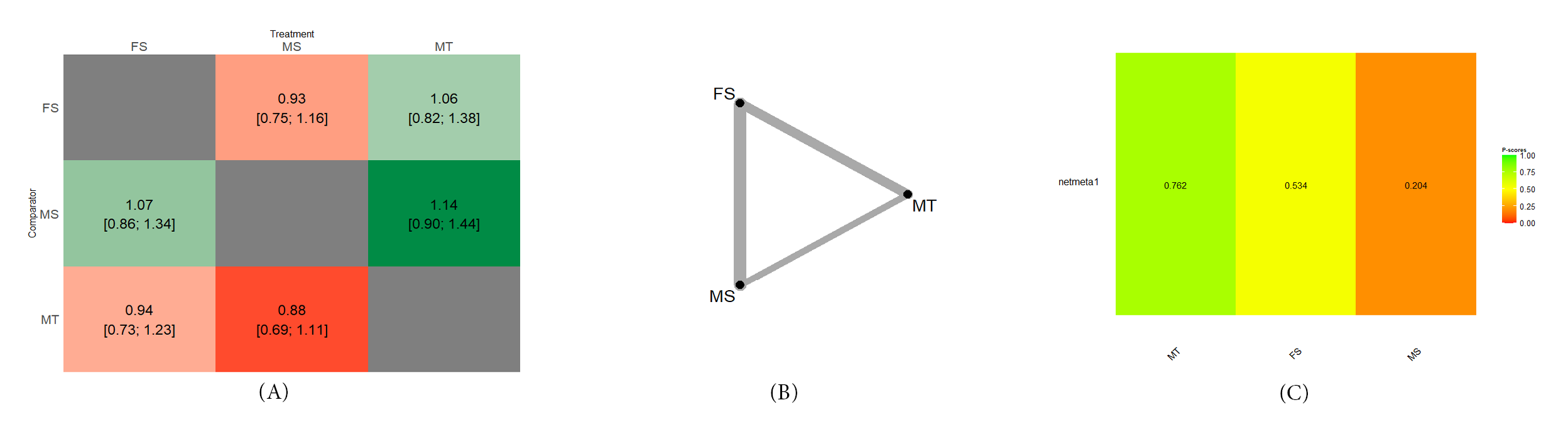


**Figure S1. NMA of atrial fibrillation.**

(A) League table plot, the number in each cell refers to the comparison between the given column and row. (B) Network plot, showing nodes that represent each approach, and edges that reflect the number of studies in each comparison. (C) P-score ranking indicating a higher ranking suggests that the specified approach was more likely to be beneficial.

FS, Full Sternotomy; MT, Mini-thoracotomy; MS, Mini-sternotomy.


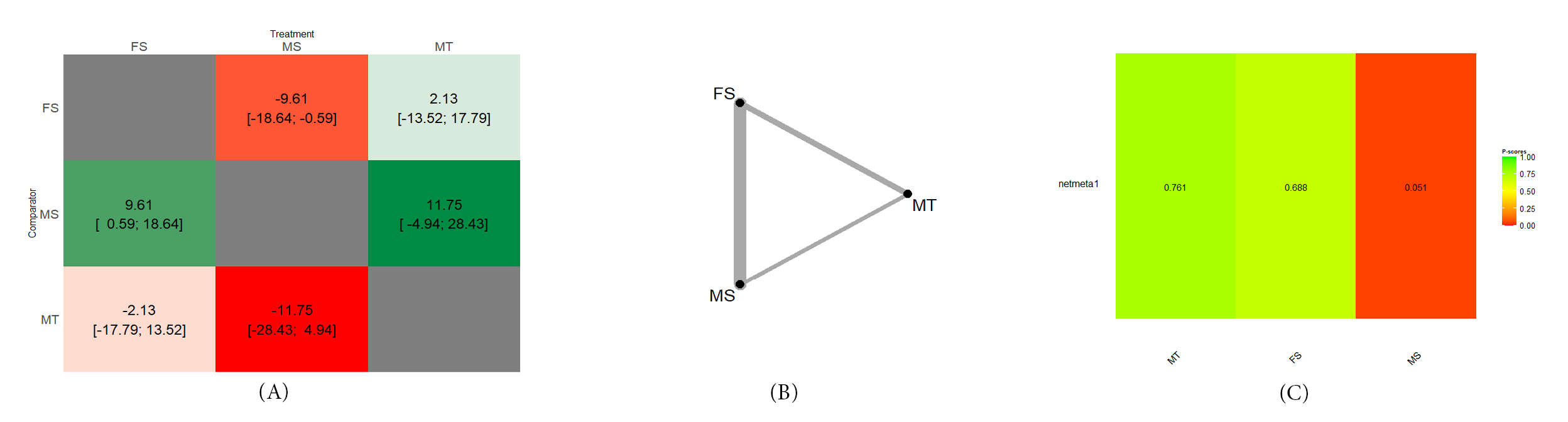


**Figure S2. NMA of bypass time.**

(A) League table plot, the number in each cell refers to the comparison between the given column and row. (B) Network plot, showing nodes that represent each approach, and edges that reflect the number of studies in each comparison. (C) P-score ranking indicating a higher ranking suggests that the specified approach was more likely to be beneficial.

FS, Full Sternotomy; MT, Mini-thoracotomy; MS, Mini-sternotomy.


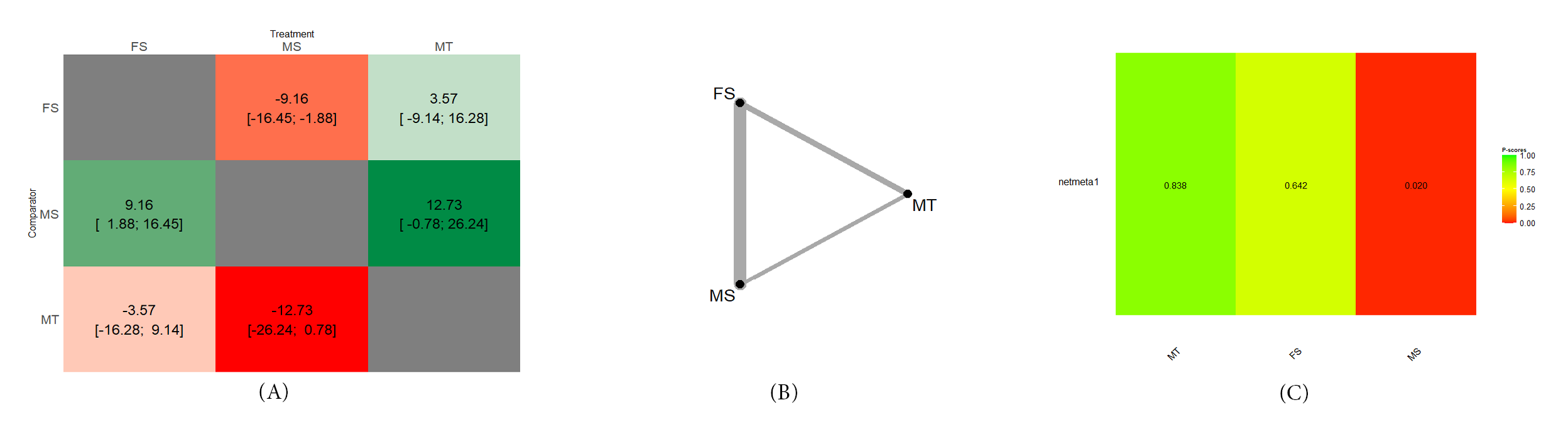


**Figure S3. NMA of** **cross-clamp time.**

(A) League table plot, the number in each cell refers to the comparison between the given column and row. (B) Network plot, showing nodes that represent each approach, and edges that reflect the number of studies in each comparison. (C) P-score ranking indicating a higher ranking suggests that the specified approach was more likely to be beneficial.

FS, Full Sternotomy; MT, Mini-thoracotomy; MS, Mini-sternotomy.


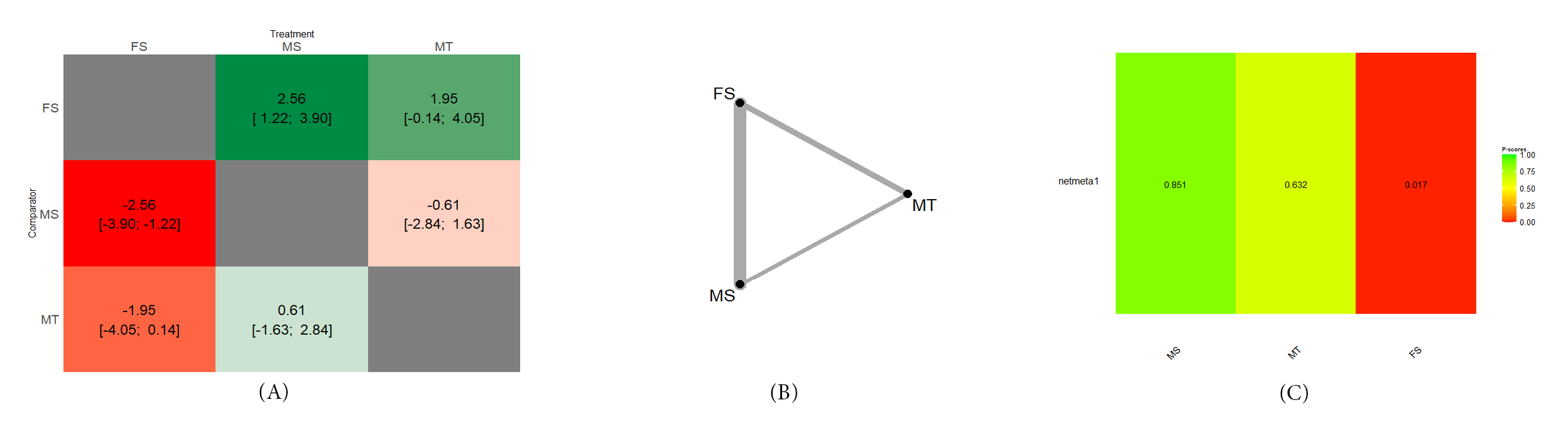


**Figure S4. NMA of** **hospital stay.**

(A) League table plot, the number in each cell refers to the comparison between the given column and row. (B) Network plot, showing nodes that represent each approach, and edges that reflect the number of studies in each comparison. (C) P-score ranking indicating a higher ranking suggests that the specified approach was more likely to be beneficial.

FS, Full Sternotomy; MT, Mini-thoracotomy; MS, Mini-sternotomy.


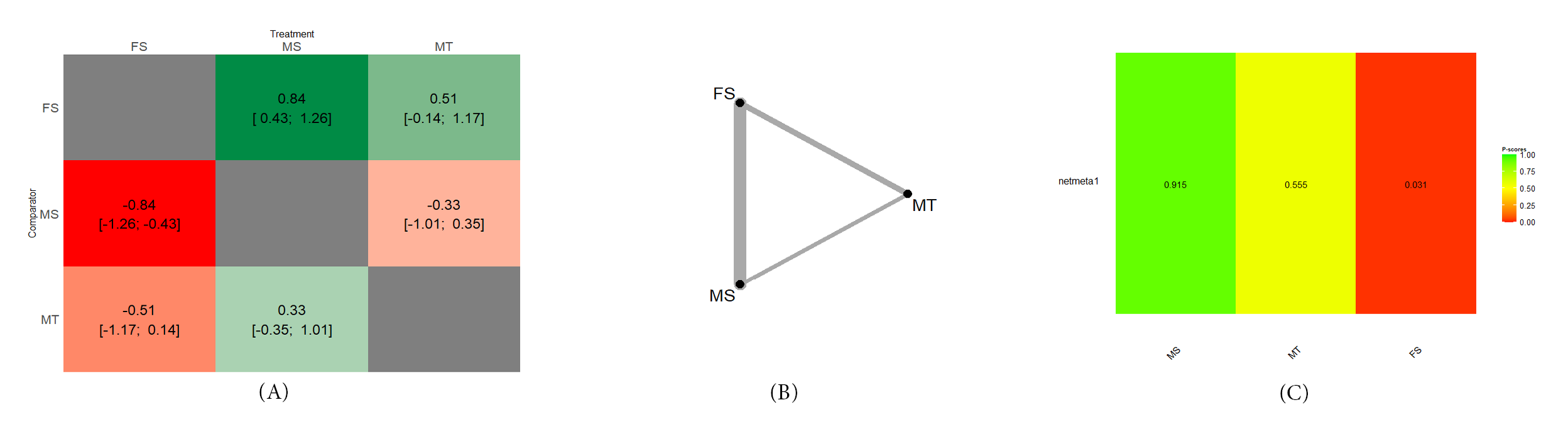


**Figure S5. NMA of** **ICU stay.**

(A) League table plot, the number in each cell refers to the comparison between the given column and row. (B) Network plot, showing nodes that represent each approach, and edges that reflect the number of studies in each comparison. (C) P-score ranking indicating a higher ranking suggests that the specified approach was more likely to be beneficial.

FS, Full Sternotomy; MT, Mini-thoracotomy; MS, Mini-sternotomy.


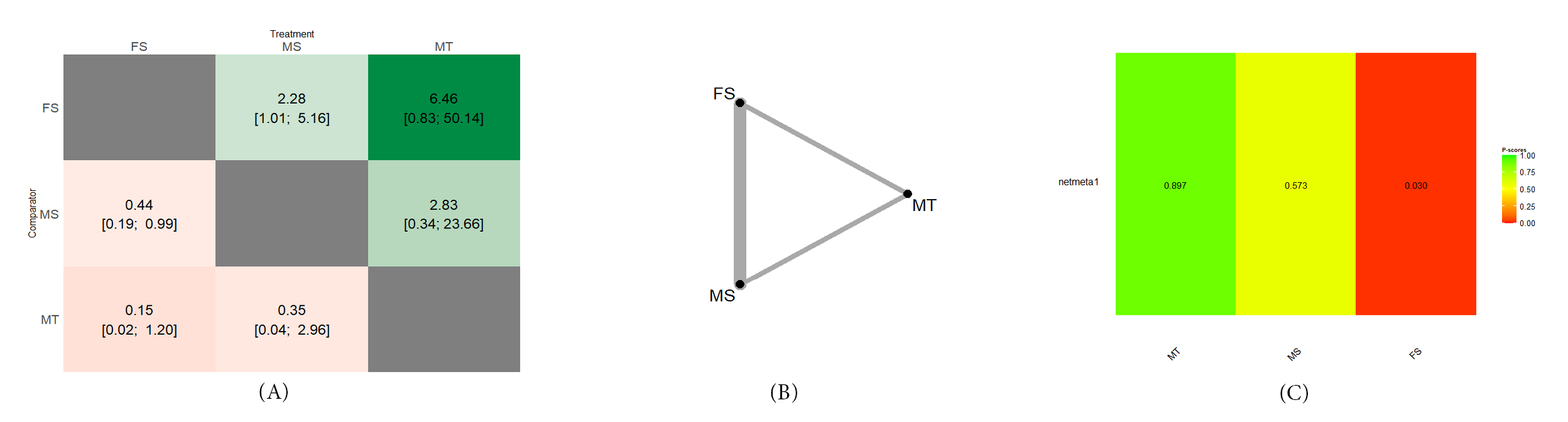


**Figure S6. NMA of** **peri-operative mortality.**

(A) League table plot, the number in each cell refers to the comparison between the given column and row. (B) Network plot, showing nodes that represent each approach, and edges that reflect the number of studies in each comparison. (C) P-score ranking indicating a higher ranking suggests that the specified approach was more likely to be beneficial.

FS, Full Sternotomy; MT, Mini-thoracotomy; MS, Mini-sternotomy.


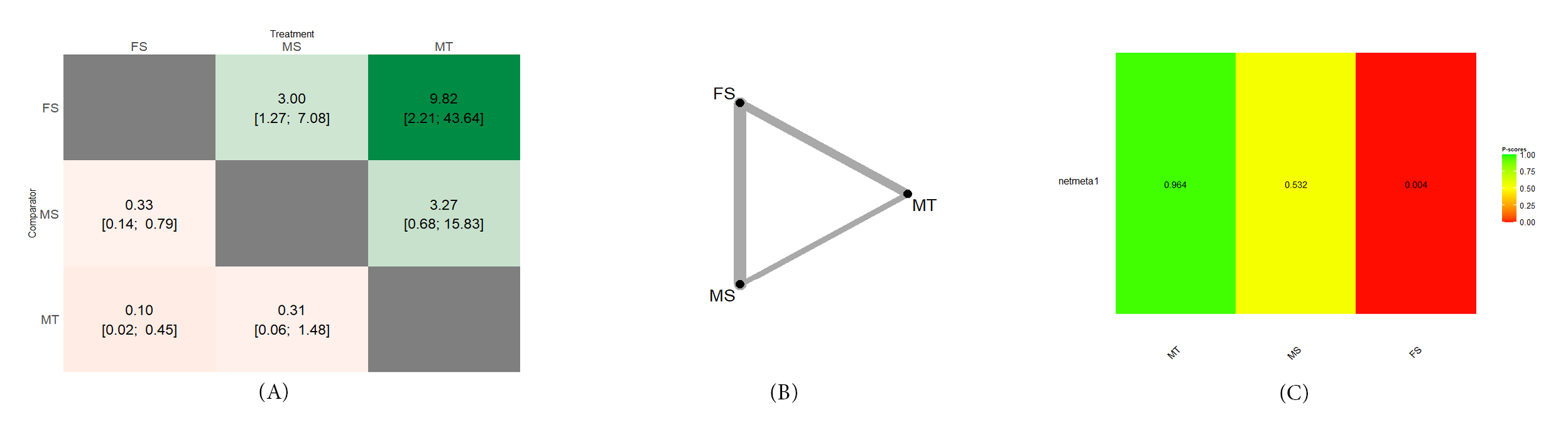


**Figure S7. NMA of** **re-exploration.**

(A) League table plot, the number in each cell refers to the comparison between the given column and row. (B) Network plot, showing nodes that represent each approach, and edges that reflect the number of studies in each comparison. (C) P-score ranking indicating a higher ranking suggests that the specified approach was more likely to be beneficial.

FS, Full Sternotomy; MT, Mini-thoracotomy; MS, Mini-sternotomy.


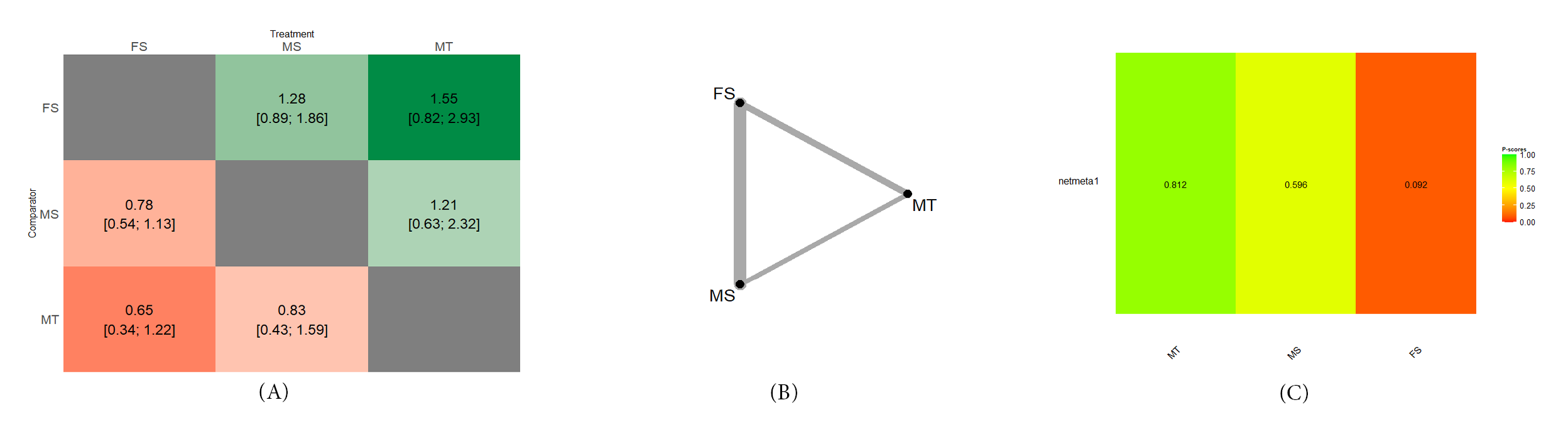


**Figure S8. NMA of** **renal failure.**

(A) League table plot, the number in each cell refers to the comparison between the given column and row. (B) Network plot, showing nodes that represent each approach, and edges that reflect the number of studies in each comparison. (C) P-score ranking indicating a higher ranking suggests that the specified approach was more likely to be beneficial.

FS, Full Sternotomy; MT, Mini-thoracotomy; MS, Mini-sternotomy.
